# Association mapping unravels the genetic basis for drought related traits in different developmental stages of barley

**DOI:** 10.1038/s41598-024-73618-y

**Published:** 2024-10-24

**Authors:** Connor Slawin, Oyeyemi Ajayi, Ramamurthy Mahalingam

**Affiliations:** 1 Cereal Crops Research Unit, USDA-ARS, 502 Walnut Street, Madison, WI 53726 USA; 2 Present Address: Department of Plant Pathology, University of Minnesota, St. Paul, MN 55108 USA

**Keywords:** Barley, Drought, Germination, GWAS, Minicore population, Polyethylene glycol, Genetics, Plant sciences

## Abstract

Drought stress significantly reduces crop yields at all stages of plant development. Barley, known for its abiotic-stress adaptation among cereals was used to examine the genetic basis of drought tolerance. A population of 164 spring barley lines was subjected to polyethylene glycol (PEG) induced drought stress during germination and seedling development. Six traits were measured, including germination percentage and rate, seedling length and weight, and root-to-shoot ratios. Seedling area, volume, and root and shoot diameter was acquired with a flatbed scanner. This population was also subjected to short-term drought during the heading stage in the greenhouse. Root and shoot weight and grain yield data were collected from well watered and droughted plants. Significant variation within traits were observed and several of them exhibited strong correlations with each other. In this population, two genotypes had 100% germination under PEG-induced drought and drought tolerance throughout the heading stage of plant development. A genome-wide association scan (GWAS) revealed 64 significant marker-trait associations across all seven barley chromosomes. Candidate genes related to abiotic stress and germination were identified within a 0.5Mbp interval around these SNPs. In silico analysis indicated a high frequency of differential expression of the candidate genes in response to stress. This study enabled identification of barley lines useful for drought tolerance breeding and pinpointed candidate genes for enhancing drought resiliency in barley.

**Supplementary Information:**

The online version contains supplementary material available at 10.1038/s41598-024-73618-y.

## Introduction

Abiotic stress is regarded as a primary threat to agriculture, potentially impacting up to 70% of the current crop yields worldwide^1^. Among the various abiotic stresses, drought is considered the most detrimental to crop productivity. Water is essential for every stage of plant development from seed germination through seed filling. Even short-term water deficits during crucial developmental transition stages like germination, seedling emergence, flowering, seed filling have an enormous negative impact on the plant stand, final biomass, seed yield, and end-use quality. Given the irregularities of precipitation in the context of the current climate-change, selection for drought tolerance is an important breeding goal for crops of economic importance.

Drought tolerance is the product of many interacting cellular responses that are dependent on the intensity and duration of water deficit^2^. Breeding for drought tolerance is challenging since it can cause dramatic changes in phenotypes required for analysis, genotype by environment interactions confounding breeding selection, and being a complex quantitative trait controlled at many loci^3^. Drought stress in early developmental stages impair seed germination and/or reduces the rate of germination^4,5^. Plant drought tolerance is determined not only by aboveground traits such as stomatal conductance or density but also belowground traits such as root hydraulic conductivity and maintenance of root growth under water stress. Roots are the key plant organ for water and nutrient uptake from soil that is directly impacted by root growth habits, architecture, distribution, structure, density, size, and proliferation. These functions are essential responses leading to adaptation under drought stress. Long, thick, and extensive root systems can uptake water from a deeper layer of soil which is inaccessible to shallower rooting vegetation. Furthermore, developed root phenotypes are considered to be an important selection criterion for drought-tolerant genotypes. However, screening for root traits in soil either in greenhouse or under field conditions requires specialized phenotyping equipment^6^ and/or is labor-intensive^7,8^.

High molecular weight polyethylene glycol (PEG) is a nonpenetrating, water-soluble and nonionic polymer that induces drought stress by causing osmotic stress and has been used extensively as an osmolyte to impose a controlled soil-free water deficit medium in various plants^9,10^ including barley^11,12^. Root traits in response to a PEG-treatment such as length, area, volume, diameter can be easily captured using a flatbed scanner. A root’s morphological measurement under PEG-induced drought stress was reported to correlate strongly with barley grain yield under drought^13^. PEG based assays are an economically viable strategy to screen many genotypes in short time^13^.

Genome-wide association study (GWAS) is an effective and powerful technique, which can associate drought resilient phenotypes with genotypes in natural populations. Associations identify natural allelic variations and candidate genes based on linkage disequilibrium (LD) and have been widely used to study crop plants^14^ and inform breeding programs. Barley is an excellent cereal model for studying the genetics of developmental, adaptive traits, and metabolite composition^15,16^ and is known for its high degree of rigor under abiotic stress^17^. GWAS has been employed to study drought tolerance in European spring barley cultivars^18^, nested association mapping population developed using wild barley accessions^19^, germplasm collection containing cultivated and wild barley accessions^20^, Ethiopian barley landraces^21^, and Tibetan wild barley accessions^22^. Through the discovery of QTL locations linked to drought tolerance, breeders can incorporate these loci into breeding programs to develop barley varieties resilient to water stress.

Although many QTLs for drought resistance have been identified through traditional linkage analysis, to date, only very few QTLs responsible for drought resistance have been cloned. In maize, a significant SNP identified by GWAS overlapped with a drought related QTL and was directly located in the *ZmVPP1* gene encoding a vacuolar-type H^+^ pyrophosphatase. In the tolerant allele of ZmVPP1 containing three MYB recognition sequences (WAACCA, W: A or T), a 366-bp insertion was found as the causative variation that conferred the drought inducible expression of ZmVPP1^23^. Furthermore, GWAS also lends itself useful to joint -omics approach, such as transcriptomics and metabolomics. From GWAS candidate gene identification, transcriptomics and differential gene expression can be used to confer and support genotypic associations^24^. In maize, a GWAS transcriptomics joint approach was used to identify *MADS*26, a transcription factor regulating seed germination^25^. Another maize GWAS transcriptomics analysis found candidate genes responsible for root architecture leading to increased drought tolerance^26^. In barley, GWAS transcriptomics projects have identified seven differentially expressed genes in response to drought stress^27^. This type of integration of genomic tools with traditional breeding approaches holds promise for accelerating the development of drought-tolerant barley cultivars^28^.

Barley (*Hordeum vulgare L.*) is an economically significant crop, with global production of 145 million metric tons, making it the fourth most important cereal^29^. In the United States, 185 million bushels of barley were produced in 2023^30^, a 6% increase from 2022. A vast majority of the barley grain serves as fodder for livestock (70%) followed by malting, brewing, and distilling processes (21%)^31^. However, the ever-shifting climate patterns, especially heat and drought stress, pose a significant threat to its productivity and negatively impact the global beer supply^32,33^. In the US, more than 80% of the spring barley growing regions were under drought in 2022. However, a 0.8 bushels per acre increase was observed despite these climatically challenging growing conditions to barley production. These production advances highlight barley’s drought tolerance over other cereals like wheat and oat which experienced yield losses over the 2022 and 2023 growing seasons^30^. Barley’s resilience in drought over other grains makes it a prime candidate for studying the genetic basis of drought tolerance and improvement for all cereals.

In this study, we leverage the power of GWAS to uncover candidate genes governing drought tolerance in barley. Utilizing the mini-core population (*n* = 155), a subset from the USDA’s barley iCore population^34^, we studied the genetic basis of drought tolerance. First, the mini-core population was phenotyped for germination performance traits and seedling development traits under chemically induced drought conditions. This population was subjected to two rounds of short-term (5-day) drought stress treatment during heading stages in greenhouse conditions. Shoot and root biomass and seed yield data were collected from control and droughted plants. QTLs associated with chemically-induced drought during germination, seedling stages, and short-term drought in heading-stage plants were identified. Based on the phenotypic responses to drought during germination, seedling, and heading stages, barley lines suitable for drought-tolerance breeding were identified. GWAS analysis recognized several strong marker-trait associations. Identified candidate genes can be utilized in marker-assisted selection and biotechnological approaches for improving drought tolerance in barley.

## Results

### Phenotypic variation for germination and seedling traits under PEG-induced drought stress

**Fig. 1:**
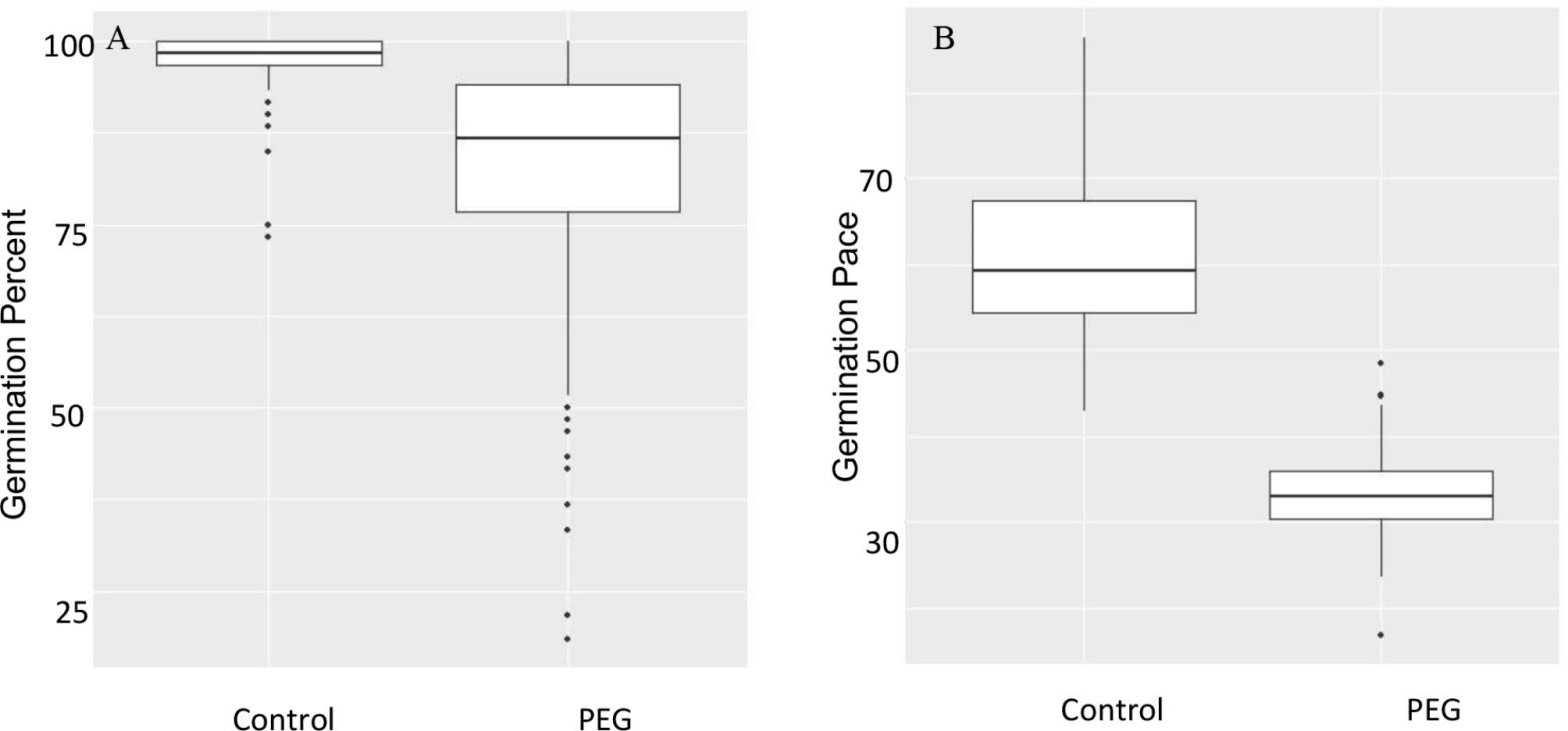
Boxplot analysis of variation of minicore population for seed traits under control and drought stress (PEG 20%). (**A**) Germination percentage; (**B**) Germination pace.

The various phenotypic traits related to seeds and seedlings, their abbreviations, and formulas to calculate the corresponding derived traits are presented in Table 1. A large amount of phenotypic variation was observed for all the traits in the mini-core collection both under control (Supplementary Table 1) and chemically induced drought (20% PEG) (Supplementary Table 2). PEG treatment caused significant negative impact on both the seed traits evaluated in this study -germination percent and germination rate (Fig. 1). There were nine accessions which exhibited 100% germination in response to PEG, while 10 accessions showed less than 50% germination under PEG conditions.

**Table 1:** List of phenotypic traits evaluated in the barley minicore population.

Trait	Name	Measurement description
G%	Germination percentage	G% = n/*N*×100, n is the number of germinated seeds after 5 days, N is the total number of seeds
GR	Germination rate	GR = N/(∑(n×g)), N is the number of germinated seeds after 5 days, n is the number of newly germinated seeds on day g, g = (1,2,3,4,5)
DTI (G%)	Drought Tolerance Index (Germination Percentage)	DTI (G%) = (G% under drought)/(G% under control)×100
DTI (GR)	Drought Tolerance Index (Germination rate)	DTI (GP) = (GP under drought)/(GP under control)×100
Reduction (G%)	Reduction (Germination Percentage)	Reduction (G%) = G% under control – G% under drought
DTI (SY)	Drought Tolerance Index (Seed Yield)	DTI (SY) = (SY(g) under drought)/(SY (g)under control)×100
RFW	Root Fresh Weight	RFW = root fresh weight (g)
SFW	Shoot Fresh Weight	SFW = shoot fresh weight (g)
RDW	Root Dry Weight	RDW = root dry weight (g)
SDW	Shoot Dry Weight	SDW = shoot dry weight (g)
RTL	Root Total Length	RTL = total root length (cm)
STL	Shoot Total Length	STL = total shoot length (cm)
RA	Root Area	RA = total root area (cm²)
SA	Shoot Area	SA = total shoot area (cm²)
RSA	Root Surface Area	RSA = total root surface area (cm²)
SSA	Shoot Surface Area	SSA = total shoot surface area (cm²)
RD	Average Root Diameter	RD = Average Root Diameter (mm)
SD	Average Shoot Diameter	SD = Average Shoot Diameter (mm)
RV	Root Volume	RV = Root Volume (cm³)
SV	Shoot Volume	SV = Shoot Volume (cm³)
RL	Root Length	RL = root length (cm)
SL	Shoot Length	SL = shoot length (cm)

**Fig. 2:**
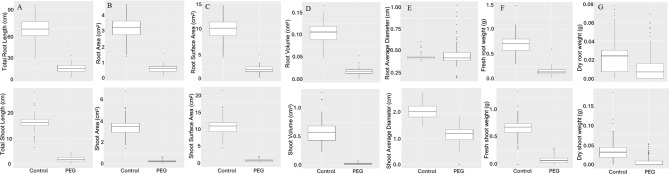
Boxplot analysis of variation in minicore population for seedling traits under control and drought stress (20% PEG). (**A**) Length; (**B**) Area; (**C**) Surface area; (**D**) Volume; (**E**) Diameter; (**F**) Fresh weight; (**G**) Dry weight. Top panel is associated with roots and bottom panel is for shoots.

All the shoot traits evaluated (Area, Diameter, Length, Volume, Weight) were significantly lower in the PEG treatment compared to the control group (Fig. 2). Similarly, all the root traits were significantly lower in the PEG treatment compared to the control group, except for root diameter. Broad sense heritability and genetic variance estimates from ANOVA are summarized in Table 2. The wide range of phenotypic variation in conjunction with the high heritability of the evaluated phenotypes lends well for the genetic dissection of these traits by GWAS.

**Table 2:** Descriptive statistics for the measured traits under control, PEG treatment and drought stress.

	Control								PEG (20%) or Drought stress							
Trait^a^	Mean	SD	SE	Variance	Min	Max	Range	H^b^	Mean	SD	SE	Variance	Min	Max	Range	H^b^
G%	96.94	± 4.02	0.32	16.15	80.00	100	20.00	0.89	81.94	± 17.09	1.37	291.99	18.33	100	81.67	0.98
GR	61.25	± 9.12	0.73	83.16	42.78	82.35	39.57	0.88	33.38	± 4.51	0.36	20.33	16.73	48.54	31.81	0.78
RTL	74.26	± 16.31	1.31	266.15	13.89	109.91	96.02	0.99	15.22	± 6.04	0.49	36.52	1.46	34.79	33.33	0.96
RA	3.15	± 0.65	0.05	0.43	0.93	4.54	3.61	0.60	0.63	± 0.22	0.02	0.05	0.10	1.56	1.46	0.96
RSA	9.89	± 2.05	0.17	4.22	2.48	14.26	11.78	0.60	1.97	± 0.70	0.06	0.49	0.31	4.90	4.59	0.96
RD	0.43	± 0.03	0.00	0.00	0.38	0.66	0.28	0.66	0.45	± 0.11	0.01	0.01	0.19	1.03	0.84	0.97
RV	0.11	± 0.02	0.00	0.00	0.03	0.17	0.14	0.62	0.02	± 0.01	0.00	0.00	0.004	0.06	0.01	0.96
STL	16.40	± 2.58	0.21	6.64	2.91	28.36	25.45	0.96	2.10	± 1.12	0.09	1.25	0.00	5.07	5.07	0.89
SA	3.42	± 0.73	0.06	1.44	0.53	6.78	6.25	0.93	0.27	± 0.14	0.01	0.02	0.00	0.65	0.65	0.89
SSA	10.74	± 2.28	0.18	5.20	1.67	21.31	19.64	0.93	0.85	± 0.42	0.03	0.18	0.00	2.04	2.04	0.89
SD	2.07	± 0.28	0.02	0.08	1.58	2.85	1.27	0.78	1.11	± 0.40	0.03	0.16	0.00	1.90	1.90	0.88
SV	0.58	± 0.18	0.01	0.03	0.29	1.28	0.99	0.87	0.03	± 0.02	0.001	0.00	0.00	0.08	0.08	0.89
RL	14.70	± 2.05	0.17	4.21	5.28	19.00	13.72	0.93	4.52	± 1.07	0.09	1.15	1.00	6.81	5.81	0.96
RFW	0.70	± 0.17	0.01	0.03	0.16	1.48	1.32	0.95	0.14	± 0.07	0.005	0.005	0.01	0.60	0.59	0.93
RDW	0.02	± 0.40	0.03	0.16	0.00	0.07	0.07	0.97	0.01	± 0.01	0.001	0.000	0.00	0.07	0.07	0.50
SL	15.12	± 2.35	0.19	5.51	2.90	23.72	20.82	0.95	1.90	± 1.08	0.09	1.16	0.00	5.00	5.00	0.87
SFW	0.67	± 0.16	0.01	0.03	0.00	1.39	1.39	0.81	0.09	± 0.07	0.005	0.004	0.00	0.30	0.30	0.94
SDW	0.04	± 0.06	0.005	0.004	0.00	0.73	0.73	0.97	0.006	± 0.01	0.00	0.00	0.00	0.06	0.06	0.66
SY	44.16	± 17.90	3.44	318.41	0.05	94.93	94.88	0.63	10.76	± 7.63	0.84	57.92	0.00	39.91	39.91	0.50

^a^Trait abbreviations are given in Table 1; ^b^Heritability.

### Phenotypic variation for short-term drought stress during heading

The measurements of root weight, shoot weight, and seed yield per plant were collected in greenhouse conditions for both irrigated plants and plants subjected to a five day drought during heading (Supplementary Table 3). Significant variation in the heading time has been observed for this population. The earliest heading was 71 days after planting, while the latest was 138 days. The median heading time was 91 days. With an arbitrary cutoff ≤ 30% yield loss after short-term water stress, five lines were identified in the mini-core collection that can be considered as potential drought tolerant lines.

### Correlation analysis

**Fig. 3:**
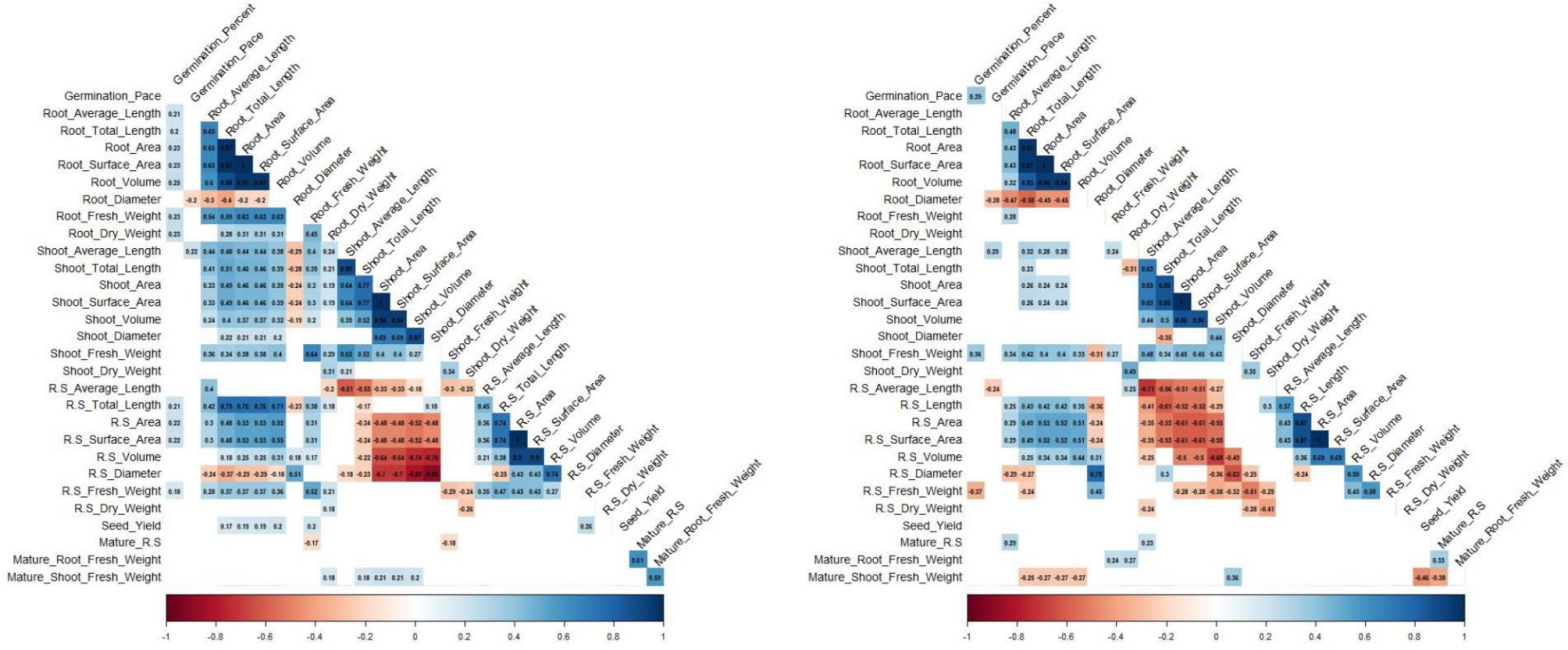
Correlation analysis of seed germination, seedling traits, and mature plant traits under control and drought stress. (**A**) Control; (**B**) PEG 20% or 5-days of drought during heading.

Correlation matrix was generated to evaluate the relationships between the various phenotypic traits evaluated in this study (Fig. 3). Significant correlations between traits in the control (Fig. 3A) and PEG/drought treatment group (Fig. 3B) were found. Closely related traits such as fresh weight, dry weight, length, area, and volume were consistently positively correlated with each other for both the roots and shoots. However, root diameter was negatively correlated with root length both in the control (-0.40) and in PEG treatment (-0.58). Shoot fresh weight and root fresh weight showed a significant positive correlation in control (0.64) and in the PEG treatment (0.27). Germination rate and root diameter showed a negative correlation in control (-0.2) and in the PEG treatment (-0.28). Germination percentage and germination rate were positively correlated (0.39) only under PEG treatment. Germination percentage showed a positive correlation with root: shoot fresh weight under control (0.22) and a negative correlation under PEG (-0.37).

**Fig. 4:**
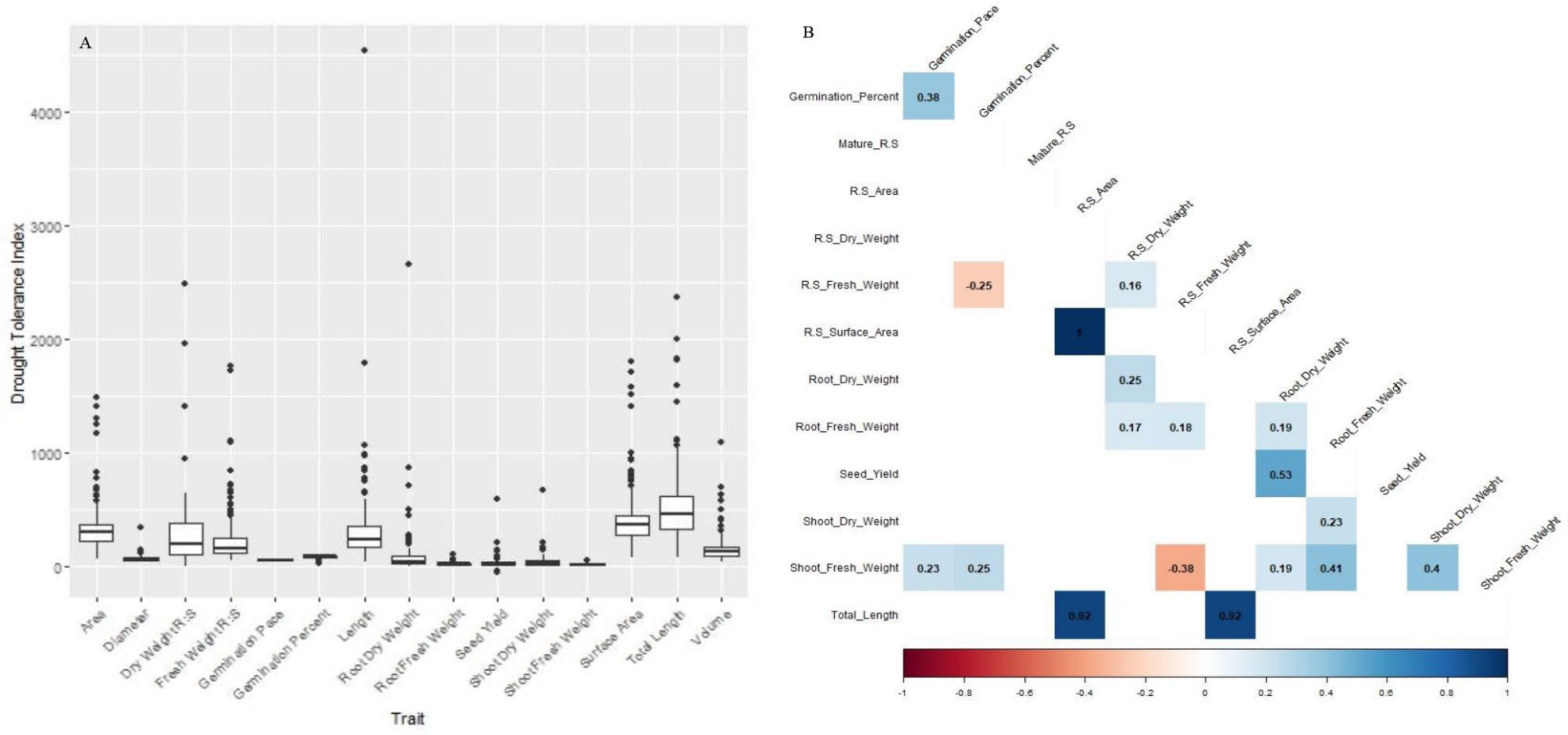
Drought tolerance index of seed germination, seedling traits and mature plant traits. (**A**) Box plot analysis. (**B**) Correlation analysis.

For Drought Tolerance Index (DTI), most correlations were positive owing to a reduction commonly seen across phenotypes in response to the PEG treatment (Fig. 4A). However, positive correlations were observed (0.38) between germination percentage and germination rate, fresh weight of shoots and germination percentage (0.25), germination pace (0.23) and fresh weight of roots (*r* = 0.41). The negative correlation (-0.38) between total fresh weight and shoot fresh weight is indicative of the strong treatment effect imposed by PEG (Fig. 4B).

The reduction correlation matrix (Supplementary Fig. 2) displayed similar patterns as the DTI matrix, with most correlations being positive. In this index, root and shoot fresh weights were again found to be positively correlated (*r* = 0.57). The negative correlation between seed yield and germination rate (-0.25) was unexpected and warrants further investigation.

Under control conditions, root: shoot weight ratios was positively correlated with root weight (0.33) and negatively correlated with shoot weight (-0.38) (Fig. 3A). Mature shoot weight was negatively correlated with seed yield (-0.48) under drought stress conditions (Fig. 3B). Interestingly, under drought stress, shoot weight was negatively correlated with root length, area, and volume (-0.27), whereas, seed yield under irrigated conditions were positively correlated with total root length (0.17), root area (0.19) and root volume (0.20). The most interesting observation was a positive correlation (0.53) between the DTI seed yield and root dry weight (Fig. 4B). Of the nine lines that exhibited 100% germination in PEG treatment, three of them maintained large root lengths in seedling stage and furthermore, two of these lines had no significant impact on seed yields in response to drought stress during heading.

### Genome wide associations

Filtering of genotypic data included using a minor allele frequency (MAF) ≥ 0.05, and minimum call rate of 80%. Following filtering, 155 accessions and 39,789 SNPs remained, of which 36,421 markers were identified as informative. SNP markers were evenly dispersed throughout the seven chromosomes, and more densely covered genomic regions with higher frequencies of recombination, such as telomeres (Supplementary Fig. 3).

**Fig. 5:**
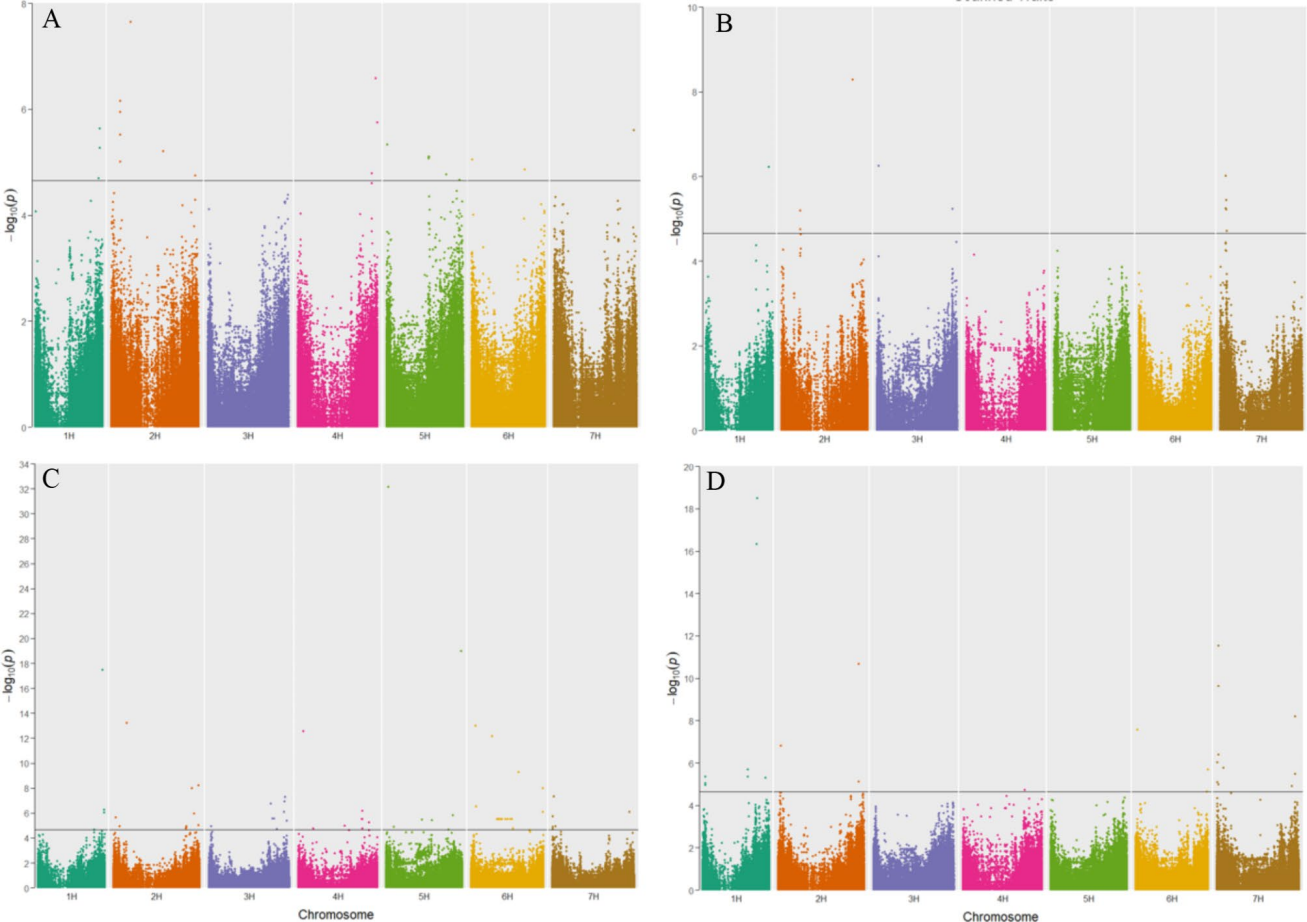
Manhattan plots of genome-wide association studies in barley minicore population using FarmCPU model. (**A**) Measured traits (**B**) Traits evaluated using the scanner (**C**) Drought Tolerance Index traits (**D**) Reduction traits. Significant SNPs are above the blue horizontal line (Y-axis: –log_10_ p-value ≥ 4.65).

**Fig. 6:**
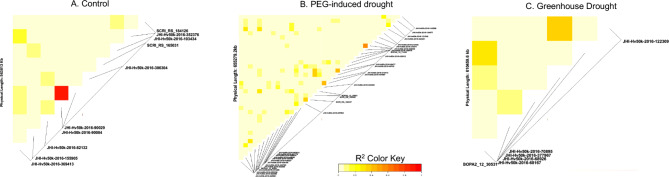
Pairwise linkage disequilibrium of identified SNPs in GWAS analysis separated by treatment group. Panel A = control group; panel B = PEG-induced treatment group; panel C = 5-day short term drought during heading stage. LD coefficients are reported as r^2^ over genetic distance in base pairs.

GWAS analysis of the 155 spring barley accessions was conducted to identify QTLs responsible for the genetic basis of germination and seedling development. The FarmCPU modeled GWAS analysis detected 224 significant marker-trait associations with a –log_10_ p-value ≥ 4.65, which was set by an FDR of 0.01 and was spread across all seven barley chromosomes (Fig. 5). The physical location of significant SNPs was used to identify candidate genes on the reference genome MorexV3 pseudomolecules assembly. SNPs which were within linkage disequilibrium (LD) ±0.5 MB window, identified via LD decay, were co-located, and reported as a single marker association (Fig. 6). Further, only those significant SNPs accounting for 5% or more of the phenotypic variance explained (PVE) were considered for further analysis. This stringent filtering criteria led to 87 high quality SNPs associations (22 for control, 51 for PEG treatment, 12 for greenhouse drought treatment) across the seven barley chromosomes (Supplementary Table 4).

Of the 22 SNPs in the control group, four SNPs were associated with two or more traits and 11 SNPs were unique to one particular trait (Supplementary Table 4). Eight SNPs were located on chromosome 2, two on chromosomes 4 and 5, and one each on chromosomes 1, 3, and 6.

For the 51 SNPs identified in association with the PEG treatment group, seven SNPs were associated with two or more traits while 31 SNPs were unique to one particular trait. In descending order, eight SNPs were localized on chromosome 5, chromosomes 2 and 7 each had seven SNPs, chromosome 6 had six SNP associations, chromosomes 1 and 3 had five SNPs, and chromosome 4 had the least with two SNPs.

Of the 12 SNPs in the greenhouse study, three SNPs were associated with two traits and six SNPs were unique to one particular trait. Five of the SNPs were on chromosome 2 and one SNP was associated each on chromosomes 4, 5, 6, and 7.

### Germination percentage

In total, five SNPs showed significant associations (–log_10_ p-value ≥ 4.65) with germination percentage trait on four different chromosomes (Table 3, Supplementary Table 4). The SNP on chromosome 6 (JHI-Hv50k-2016-369413) identified in control, was the most significant association accounting for 88.6% of PVE. The two significant SNPs under induced drought were on chromosomes 5 (JHI-Hv50k-2016-134977) and 2 (JHI-Hv50k-2016-310413), accounting for 17.5% (p-value = 1.72 e^− 5^) and 9.3% (p-value = 1.8e^− 5^), respectively, for the variation in germination percentage. One SNP (JHI-Hv50k-2016-420812) on chromosome 6 was associated with DTI of germination percentage, explaining 27.1% of the variation (p-value = 1.52e^− 5^) and with reduction of germination percentage trait explaining 26.5% phenotypic variance (p-value = 1.77e^− 5^).

**Table 3:** Significant SNPs associated with drought-related traits that are localized within predicted genic regions of barley genome.

SNP (Control)	Ch	SNP Pos	*P*-value	MAF	Traits^a^	PVE	GeneID^b^	SNP location	BARLEX gene description
JHI-Hv50k-2016-45487	1 H	484,160,623	2.02E-05	0.08	RDW	8.7	1HG0079630	Third intron	Sec14p-like protein
JHI-Hv50k-2016-82132	2 H	67,370,194	3.01E-06	0.12	SFW	14.3	2HG0119530	Third intron	Stomatal closure-actin-binding protein
JHI-Hv50k-2016-90004	2 H	146,167,997	1.83E-05	0.21	RTL	10.8	2HG0132140	fifth intron	Importin 5
JHI-Hv50k-2016-90029	2 H	146,772,819	2.27E-08	0.20	SL	7.8	2HG0132190	sixth intron	DNA repair protein-like protein
JHI-Hv50k-2016-103434	2 H	551,327,437	2.51E-08	0.37	SL	7.0	2HG0180040	first intron	Trichome birefringence
JHI-Hv50k-2016-155905	3 H	12,515,587	5.64E-07	0.14	SA	11.9	3HG0224880	first intron	NBS-LRR gene
SCRI_RS_165031	4 H	523,178,575	1.04E-05	0.26	SL	7.9	4HG0394970	first intron	Allene oxide synthase
SCRI_RS_184126	4 H	566,574,431	2.49E-05	0.12	RDW	32.1	4HG0404040	Second exon	Succinate dehydrogenase factor 1
JHI-Hv50k-2016-306304	5 H	419,551,108	2.19E-05	0.10	RL	12.0	5HG0478580	fourth exon	Inositol-1,4,5-trisphosphate-phosphatase
JHI-Hv50k-2016-352376	5 H	564,102,809	5.63E-09	0.24	SL	7.6	5HG0526670	exon	Leucine-rich repeat receptor kinase
JHI-Hv50k-2016-369413	6 H	2,761,416	8.92E-06	0.10	G%	88.6	6HG0539150	500 bp up	Retinoic acid-neural-specific protein
**SNP (PEG-treatment)**									
JHI-Hv50k-2016-33403	1 H	417,903,074	4.70E-17	0.05	Red: RDW	37.0	1HG0063340	3.5 kb down	B-cell receptor-associated 31-like
JHI-Hv50k-2016-36022	1 H	434,703,493	2.15E-05	0.07	DTI: SFW	20.0	1HG0066520	third intron	magnesium transporter, (DUF803)
JHI-Hv50k-2016-49477	1 H	495,000,177	2.33E-06	0.45	SFW	30.1	1HG0084970	fifth intron	Phosphatidylserine synthase 2
JHI-Hv50k-2016-49703	1 H	495,246,916	1.26E-05	0.44	RD	12.3	1HG0085090	2863 bp up	F-box protein
BOPA2_12_11449	2 H	555,965,096	1.85E-05	0.08	DTI: RFW	7.1	2HG0181020	Exon 1	Tetratricopeptide repeat-like protein
BOPA2_12_20326	2 H	46,947,830	1.16E-05	0.10	DTI: SFW	10.4	2HG0114290	second intron	Xylose isomerase
JHI-Hv50k-2016-121548	2 H	619,749,368	2.15E-11	0.06	Red: RDW	26.2	2HG0197970	1.7 kb down	von Willebrand factor A domain protein
JHI-Hv50k-2016-134977	2 H	643,186,114	1.80E-05	0.17	G%	9.3	2HG0208270	7th exon	Superoxide dismutase
JHI-Hv50k-2016-143568	2 H	657,287,526	9.56E-06	0.11	DTI: RL	7.0	2HG0214820	12th exon	Protein kinase
JHI-Hv50k-2016-70142	2 H	18,242,220	2.26E-06	0.06	DTI: RFW	12.7	2HG0104650	first intron	Thioredoxin
JHI-Hv50k-2016-158478	3 H	17,213,010	1.21E-05	0.10	DTI: RL	6.7	3HG0227570	5th exon	Calcineurin B-like protein
JHI-Hv50k-2016-206096	3 H	568,001,594	4.74E-06	0.37	Red: GR	9.1	3HG0308720	1st intron	BSD domain protein
JHI-Hv50k-2016-210371	3 H	582,543,356	9.88E-09	0.09	RL	14.0	3HG0313320	3rd exon	Potassium transporter
JHI-Hv50k-2016-212430	3 H	586,502,834	5.32E-08	0.09	DTI: RL	14.5	3HG0314840	6.35 kb up	P-loop containing NTP hydrolase
JHI-Hv50k-2016-223231	3 H	612,137,874	5.58E-08	0.07	SD	25.7	3HG0327190	5th intron	villin
BOPA2_12_20831	4 H	355,873,157	1.08E-05	0.17	DTI: RFW	6.5	4HG0374910	14th intron	Beta-adaptin-like protein
JHI-Hv50k-2016-225661	4 H	2,008,357	2.28E-05	0.09	RD	12.0	4HG0331940	6th exon	Copper ion-binding protein
BOPA2_12_30531	5 H	10,112,592	3.79E-06	0.10	RD	18.5	5HG0424720	2nd exon	DNA polymerase II large subunit|
JHI-Hv50k-2016-297664	5 H	276,270,519	3.59E-06	0.25	DTI: RDW	58.6	5HG0459090	2nd exon	R3H domain-containing protein 4
JHI-Hv50k-2016-310413	5 H	459,413,788	1.72E-05	0.07	G%	17.5	5HG0486370	2.2 kb down	Remorin
JHI-Hv50k-2016-352386	5 H	564,103,552	8.54E-06	0.23	DTI: GR	11.0	5HG0526670	Exon 1	Leucine-rich repeat receptor-like kinase
JHI-Hv50k-2016-353238	5 H	564,785,857	8.01E-06	0.31	DTI: GR	5.5	5HG0527150	2nd exon	Fructokinase
SCRI_RS_133042	5 H	351,376,321	4.02E-06	0.46	DTI: RFW	5.3	5HG0468320	exon 1	Inosine-5’-monophosphate dehydrogenase
SCRI_RS_182407	5 H	325,230,462	7.82E-06	0.41	SDW	10.4	5HG0464670	exon 2	Long-Chain Acyl-CoA Synthetase
JHI-Hv50k-2016-374365	6 H	13,433,783	6.71E-06	0.08	RL	8.9	6HG0543700	exon 6	ATP-dependent helicase family protein
JHI-Hv50k-2016-404363	6 H	404,580,149	1.36E-05	0.08	RFW	46.2	6HG0599830	intron 1	WRKY TF
JHI-Hv50k-2016-407667	6 H	453,226,671	2.41E-05	0.05	DTI: RFW	28.7	6HG0605750	exon 2	N-acetyltransferase, putative
JHI-Hv50k-2016-420812	6 H	533,706,095	1.52E-05	0.50	DTI: G%	27.1	6HG0622520	65 bp up	Amino acid transporter family protein|
JHI-Hv50k-2016-432785	6 H	559,301,333	2.09E-06	0.05	Red: RDW	6.5	6HG0633000	exon	3-ketoacyl-CoA synthase
JHI-Hv50k-2016-438195	7 H	3,481,866	7.95E-06	0.05	Red: SDW	68.4	7HG0635790	intron 6	Arginine N-methyltransferase protein
JHI-Hv50k-2016-438327	7 H	3,494,252	4.91E-06	0.10	RL	7.5	7HG0635820	intron 11	SEC16B-like protein
JHI-Hv50k-2016-439099	7 H	4,190,161	9.42E-07	0.07	Red: SFW	5.5	7HG0636350	exon 4	Chorismate synthase
JHI-Hv50k-2016-445646	7 H	10,981,309	2.92E-12	0.06	Red: RDW	18.3	7HG0640390	exon 2	Flavin-containing monooxygenase
JHI-Hv50k-2016-459904	7 H	42,645,224	9.66E-07	0.30	RV	8.6	7HG0654060	exon 4	Hexosyltransferase
JHI-Hv50k-2016-461178	7 H	47,461,380	2.00E-05	0.41	RL	27.5	7HG0655770	intron 11	Early Nodulin 93
JHI-Hv50k-2016-462613	7 H	50,952,303	1.67E-06	0.09	Red: SFW	5.4	7HG0656850	3.3 kb down	AT hook, DNA-binding protein
**SNP (Greenhouse)**									
JHI-Hv50k-2016-100142	2 H	523,925,893	4.18E-06	0.06	GRed_R: S	5.7	2HG0175000	657 bp up	LRR kinase-like
JHI-Hv50k-2016-68926	2 H	16,977,407	3.68E-06	0.11	GDTI_R: S	6.6	2HG0103870	9th intron	Vacuolar sorting protein 33
JHI-Hv50k-2016-122309	2 H	620,572,219	3.76E-06	0.31	GDTI_R: S	8.8	2HG0198330	intron 1	Serine hydroxymethyltransferase
JHI-Hv50k-2016-70895	2 H	19,839,496	2.16E-11	0.06	GRed: SY	24.9	2HG0105180	exon 2	PHD finger protein
JHI-Hv50k-2016-68167	2 H	15,326,587	2.47E-05	0.12	G_SDW_C	27.8	2HG0102690	exon 2	Translation initiation factor IF-1
BOPA2_12_30531	5 H	10,112,592	1.03E-09	0.09	GRed_SY	11.8	5HG0424720	2nd exon	DNA polymerase II large subunit
JHI-Hv50k-2016-377967	6 H	18,102,214	6.22E-06	0.49	G_RDW_C	29.8	6HG0546810	818 bp down	Pentatricopeptide repeat protein

^a^Trait abbreviations are explained in Table 1. Red: Reduction trait index for PEG treatment; GRed: Reduction trait index for greenhouse experiment; GDTI: drought tolerance index for greenhouse.

^b^Each of the Geneids must be preceded by HORVU.MOREX.r3. to reflect the version 3 genome annotations based on the Morex barley genome.

### Germination rate

For germination rate, all three SNPs identified were observed in response to PEG treatment. Two SNPs (JHI-Hv50k-2016-353238 and JHI-Hv50k-2016-352386) in proximity on chromosome 5 were associated with DTI, explaining 10.9% and 5.5% of the variation, respectively. One SNP (JHI-Hv50k-2016-206096) on chromosome 3 associated with the reduction of germination rate, accounted for 9.1% of the variation in this trait (p-value = 4.74e^− 6^) under PEG-induced drought.

### Length

Twenty significant SNPs were associated with various root and shoot length traits. These associations were made across all barley chromosomes, except chromosome 1. Of the 20 SNPs, nine were associations with the control group and 11 were in response to PEG-induced drought.

#### Root length

For root length, three SNPs were identified, of which, two SNPs were associated under control conditions and one SNP under induced drought. The SNP (JHI-Hv50k-2016-90004) on chromosome 2 explained 10.8% of the variation (p-value = 1.83e^− 5^) and the second SNP (JHI-Hv50k-2016-306304) on chromosome 5 accounted for 12% of the variation (p-value = 2.19e^− 5^). The SNP (JHI-Hv50k-2016-438327) on chromosome 7 explaining 27.5% of the variation in root length (p-value = 2e^− 5^) was identified in the PEG treated group.

#### Shoot length

The one SNP (JHI-Hv50k-2016-103434) on chromosome 2 found in relation to total shoot length in the control group explained 7% of the variation in this trait (p-value = 2.51e^− 8^). For the shoot length trait, two SNPs were identified under control conditions, and both were located on chromosome 2. One of the SNPs (JHI-Hv50k-2016-90029) explained 7.8% of the phenotypic variance (p-value = 2.27e^− 8^), while the second SNP (JHI-Hv50k-2016-93576) accounted for 10.7% of the variation in shoot length, albeit at a lower significance (p-value = 6.26e^− 6^).

#### Root: shoot length

Among secondary traits associated with length, 14 SNPs were associated with the root-to-shoot ratios. For this trait’s associations, two SNPs (JHI-Hv50k-2016-438327 and JHI-Hv50k-2016-143568) identified from the PEG treatment were on chromosomes 7 and 2. These two SNPs explain 14.2% (p-value = 5.59e^− 6^) and 12.5% (p-value = 6.42e^− 6^) of the phenotypic variance associated with root: shoot total length, respectively. Furthermore, these two SNPs were associated with more than one trait. The next three SNPs were associated with root: shoot ratios for the total length, one SNP was correlated with the control group and two in the PEG treated group. The single SNP identified in the control group (JHI-Hv50k-2016-94418) was located on chromosome 2 and accounted for 11.6% PVE (p-value = 2.19e^− 5^). Six SNPs related to length were associated with average root-to-shoot measurements. Interestingly, the three SNPs identified in control group and three SNPs in the PEG treatment group were all found on different chromosomes. The three control group SNPs were located on chromosome 2 (JHI-Hv50k-2016-103250), 4 (SCRI_RS_165031), and 5 (JHI-Hv50k-2016-352376). While the simulated drought SNPs were identified on chromosome 3 (JHI-Hv50k-2016-210371), 6 (JHI-Hv50k-2016-374365), and 7 (JHI-Hv50k-2016-438327) .

#### DTI-Length

Five SNPs were associated with different DTI length traits. Three SNPs were found in relation to the DTI of total length root-to-shoot ratios on chromosomes 2, 3, and 7. The most significant SNP of these three (JHI-Hv50k-2016-438327) explained 8.7% PVE (p-value = 1.9e^− 6^), followed by (JHI-Hv50k-2016-143568) 7% PVE (p-value = 9.79e^− 6^), and the final SNP on chromosome 3 (JHI-Hv50k-2016-158478) explained 6.7% of the variation (p-value = 1.21e^− 5^). The other two DTI associated SNPs were for average root length. Both SNPs were localized on chromosome 3, the SNP (JHI-Hv50k-2016-212430) explained 14.5% (p-value = 5.32e^− 8^) and the second SNP (JHI-Hv50k-2016-210371) accounted for 6% of the variation (p-value = 1.23e^− 8^).

### Area

Six significant SNP associations were identified for the area traits of roots and shoots. As expected, the genomic regions associated with the area and surface area traits of roots and shoots overlapped.

#### Root area

Two SNPs were found in association with the root area trait. The SNP (JHI-Hv50k-2016-90004) associated with the control group’s root area and surface area localized on chromosome 2 and explained 23.9% phenotypic variance (p-value = 6.41e^− 6^). The second associated SNP (JHI-Hv50k-2016-461178) was identified in the PEG treated group, and was located on chromosome 7 explaining 28.3% phenotypic variance (p-value = 1.93e^− 5^).

#### Shoot area

For the shoot area trait, two significant SNP associations were determined. The first SNP in association returned the highest significance level for the control group (p-value = 5.32e^− 9^). This highly significant SNP (JHI-Hv50k-2016-103200) was on chromosome 2 and explained 5.5% phenotypic variance for shoot area and surface area. The second SNP (JHI-Hv50k-2016-155905) found in association with the control group shoot area and surface area was located on chromosome 3 with a PVE of 11.9% (p-value = 5.64e^− 7^).

#### Root: shoot area

Two SNPs related to root: shoot area ratios were identified. The first SNP (JHI-Hv50k-2016-103250) on chromosome 2 was associated with the control group for the area and surface area of R: S. This first SNP accounted for 12.4% PVE (p-value = 1.01e^− 7^). The next SNP (JHI-Hv50k-2016-143568) on chromosome 2 associated with the DTI of biomass area and surface area in the R: S ratio. The second SNP accounted for 31.5% (p-value = 1.01e^− 5^) of the variance and was also associated with the trait DTI root: shoot length.

### Diameter

Six SNPs were identified in relation to the average diameter of seedlings. Five associations occurred within the induced drought group. Two SNPs (JHI-Hv50k-2016-225661 and BOPA2_12_30531) associated with average root diameter were located on chromosomes 4 and 5 explaining 12% (p-value = 3.79e^− 6^) and 18.5% phenotypic variance (p-value = 2.28e^− 5^), respectively. The third SNP (JHI-Hv50k-2016-223231) associated with the average diameter of shoots on chromosome 3 accounted for 25.7% of the variation in this trait (p-value = 5.58e^− 8^). The two SNPs associated with root: shoot ratio diameter was localized on chromosome 1, one was related to average measurement and the other in reduction of diameter ratios. The SNP associated with average R: S diameter (JHI-Hv50k-2016-49703) explained 12.3% PVE (p-value = 1.26e^− 8^) and the other SNP associated with reduction in R: S diameter (JHI-Hv50k-2016-14614) accounted for 16.9% PVE (p-value = 4.55e^− 6^).

### Volume

Two SNPs were associated with root volume, one each in control and PEG treated group. The SNP (JHI-Hv50k-2016-90004) on chromosome 2 explained 36.8% phenotypic variance (p-value = 1.81e^− 5^) and was identified in the control group. The second SNP (JHI-Hv50k-2016-459904) located on chromosome 7 (8.6% PVE; p-value = 9.66e^− 7^) was identified in the PEG-treated group.

### Weight

Most of the SNP associations were identified in relation to root and shoot weight traits. Of the 28 SNPs, 16 SNP associations were related to fresh weights and 12 to dry weights.

#### Fresh weight

For fresh weights, one SNP (JHI-Hv50k-2016-404363) was associated with root weight, two SNPs (JHI-Hv50k-2016-49477 and JHI-Hv50k-2016-82132) for shoot weight, two SNPs (BOPA2_12_20831 and BOPA2_12_11449) for R: S weight, three SNPs (JHI-Hv50k-2016-70142, JHI-Hv50k-2016-407667, and JHI-Hv50k-2016-291104) for the DTI of root weight, two SNPs (BOPA2_12_20326 and JHI-Hv50k-2016-36022) for the DTI shoot weight, three SNPs (SCRI_RS_133042, BOPA2_12_20831, and BOPA2_12_11449) for the DTI of R: S, one SNP (JHI-Hv50k-2016-71911) for the reduction of root weight, and two SNPs (JHI-Hv50k-2016-462613 and JHI-Hv50k-2016-439099) for reduction of shoot weight. All SNPs that were not DTI or reduction calculations were found in the PEG-treatment group except for one SNP (JHI-Hv50k-2016-82132), which was in the control group and associated with shoot weight. Five SNPs were localized on chromosome 2, two SNPs each on chromosomes 1,4,5,6, and 7. The most significant (p-value = 9.42e^− 7^) SNP associated with reduction of shoot weight was located on chromosome 7 and explained 5.5% of the phenotypic variance associated with this trait.

#### Dry weight

SNPs associated with dry weights were distributed on six chromosomes. This included two SNPs (JHI-Hv50k-2016-45487 and SCRI_RS_184126) for root weight in the control group, one SNP (SCRI_RS_182407) for shoot weight in PEG treated group, a SNP (JHI-Hv50k-2016-297664) for the DTI of R: S weight, four SNPs (JHI-Hv50k-2016-33403, JHI-Hv50k-2016-445646, JHI-Hv50k-2016-121548, JHI-Hv50k-2016-432785) for reduction of root weight, and one SNP (JHI-Hv50k-2016-438195) for the reduction of shoot dry weight.

### Short-term drought in greenhouse

For the five-day terminal drought stress experiments, 23 significant SNP associations were identified that spanned all seven chromosomes. Twelve of these SNPs accounted for more than 5% of the variation in the traits. In this group of 12, seven SNPs were on chromosome 2, two SNPs on chromosome 4, and one SNP each on chromosomes 5, 6, and 7 (Table 3). Three SNPs (JHI-Hv50k-2016-70895, JHI-Hv50k-2016-250342, BOPA2_12_30531) associated with seed yield reduction in response to drought were found on chromosomes 2, 4, and 5, and explained 25%, 10.4% and 11.8%, respectively. Two SNPs on chromosome 2 (JHI-Hv50k-2016-68926 and JHI-Hv50k-2016-122309) were associated with the DTI of root: shoot weight. Two additional SNPs (JHI-Hv50k-2016-100142 and JHI-Hv50k-2016-68926) were associated with a reduction in root: shoot weight. Three SNPs were identified in the control plants (JHI-Hv50k-2016-68167 on chromosome 2, JHI-Hv50k-2016-250342 on chromosome 4, JHI-Hv50k-2016-489288 on chromosome 7) that were associated with shoot weight, seed yield, and root weight, respectively. The final two SNPs (JHI-Hv50k-2016-122309 and JHI-Hv50k-2016-377967) were associated with root: shoot weight ratios and root dry weight. Both SNPs were identified in drought-stressed plants and accounted for 28% and 30% of the variation in these traits, respectively.

### In silico gene expression of candidate genes

Genes located within the ± 0.5 Mbp window of LD of each significant marker were extracted identifying a total of 1446 genes. To reduce the number of genes for further consideration we concentrated on SNPs that were located inside the predicted genes and within their putative promoter regions (2 Kb) (Table 3). This caveat drastically reduced the potential candidate genes to 54. Most of the 54 genes showed differential expression (2-fold) in the 16 different tissue types that were reported (Supplementary Table 5). Fifteen genes were differentially expressed in the seedling stage comparisons involving nine different barley genotypes. In the comparison of three tissue types- radicle, plumule, and scutellum across seven different timepoints during seed germination, more than 25 of the potential candidate genes were differentially expressed. This finding further corroborates the significant marker-trait associations identified in this GWAS. The most interesting differences for the candidate genes were identified in the transcriptome analysis involving drought treatments. This involved three different studies wherein comparisons were made between drought tolerant and drought sensitive lines. Twenty genes were identified as being differentially expressed between the drought stressed and control samples (Supplementary Table 5).

## Discussion

The barley mini-core collection consisting of 155 spring barley accessions used in this study represents a subset of the USDA’s iCore collection that captures the latter’s geographic and genetic diversity^15^. The mini-core accessions exhibited a broad range of phenotypic variation across many traits during seed germination and seedling development.

The water potential of the root environment greatly impacts plant growth. In laboratory conditions the root environment can be manipulated by using osmotic solutions of polyethylene glycol (PEG). The reduction in root growth using PEG has been demonstrated successfully over the last 60 years in different plant species^35–38^. In a preliminary study with five accessions using three different concentrations of PEG (15%, 20% and 25%) it was observed that the lowest concentration did not alter the germination percentage among the accessions while the highest concentration negatively impacted germination of all the accessions (data not shown). The 20% (w/v) PEG-induced drought treatment which translates to an osmotic potential level of -1.09 MPa resulted in significant variation in germination performance (Supplementary Fig. 1) and seedling development phenotypes and was also used in other barley studies^11,12^.

Most measured traits were negatively impacted in response to PEG treatment. Interestingly, the root diameter in PEG treatment showed an increase in several accessions even though the mean difference between the control and PEG root diameters were not significantly different. Increases in diameter of roots growing against osmotic stress have been reported in maize, pine, and orange seedlings^35,39,40^ This is of interest because such increases in root diameter occur in plants growing in soils with high mechanical strength and has been postulated that the radial thickening aids roots penetrate compact soils^38,41–43^. If responses to osmotic stress can be related to mechanical stress, then PEG could be used as proxy to screen plant species for their ability to penetrate compact soils.

The correlation matrix generated in this study revealed insightful relationships between various phenotypic traits under both control and drought conditions. These correlations further highlight the complexity and quantitative nature of drought tolerance responses. Notably, the consistent positive correlations observed among closely related biomass traits such as fresh weight, dry weight, length, area, and volume for both roots and shoots emphasize the synchronized nature of germination and seedling development. In similar barley PEG-induced studies, a positive correlation between these developmental growth and germination traits has been identified^12,44^. The negative correlation between root diameter and root length in both control and PEG-treated plants presents a possible trade-off mechanism where increases in one dimension may come at the expense of the other dimension. Furthermore, this dynamic suggests an adaptive strategy to optimize growth and photosynthate resource use under varying water availability. This tradeoff between root diameter and root length was also observed in greenhouse conditions^45^. The potential for a growth fitness model under drought conditions is worthy of further investigation.

Drought tolerance is a complex quantitative trait which is impacted by the stage of plant development, as well as the duration and severity of the stress. Nonetheless, the negative effects of drought stress during all growth stages from seed germination to seed setting must be considered given the vagaries due to climate change. Drought stress can not only inhibit germination and seedling establishment, but it also has significant adverse effects on seed yield and quality in barley^46^. The negative correlation between mature plant shoot weight and seed yield is consistent with our recent QTL mapping of shoot weight trait in response to drought stress in a recombinant inbred line population^47^. Higher shoot weight in response to drought indicates inefficient translocation of the stem assimilates to the developing grain leading to reduced seed yield and seed weight^3,48^. The combination of PEG-based screening of seedlings and the short-term drought stress in adult plant stage in this study enables to answer the central hypothesis of the reliability of proxy methods in identifying drought tolerant lines. Among the high performing lines, MC 147 in terms of root length and MC 180 and MC 004 with reference to germination pace under PEG treatment were also found to have low shoot weight and produced higher seed yield per plant under drought stress. The consideration of germination rate and root length traits along with germination percentage in the PEG and control groups, will ensure selecting all the potential lines that were identified are drought tolerant based on our greenhouse experiment.

Significant unique SNPs (65) were identified across all the seven chromosomes in this study. About 30% of these SNPs were on Chromosome 2 (20) followed by chromosome 5 (11 SNPs). In another PEG based screening study, three regions of the barley chromosome 2 were reported to bear significant SNPs associated with shoot length, root length, and germination rate traits^12^. A meta-analysis of QTLs associated with abiotic stress tolerance in barley identified chromosome 2 to harbor 34 drought responsive QTLs, 29 salinity related QTLs, and 25 QTLs related to waterlogging. The same study also indicated chromosome 5 harbored 36 salinity QTLs, 19 associated with low temperature stress, and 12 QTLs related to drought^49^. The presence of QTLs associated with different abiotic stresses on a chromosome are a strong indicator of the presence of major genes impacting multiple related traits. Such chromosomal regions can vastly enhance selection efficiency for developing climate-resiliency when incorporated into marker assisted breeding programs.

In recent years the wide use of GWAS to uncover genomic regions associated with PEG-induced drought stress has been reported in several crops including barley^12,50–53^. The availability of a well annotated barley genome enabled precise localization of the SNPs. Four significant SNPs from the control group, 15 from the PEG treatment group, and three SNPs identified in the greenhouse drought experiment that were localized in exonic regions were considered as candidate genes. These exonic SNPs were associated with genes enriched for the gene ontology of redox homeostasis biological process. These genes were involved in four major biological processes – stress and hormone signaling, lipid metabolism, DNA metabolism, and mitochondrial activities.

Leucine Rich Receptor **(**LRR) kinase gene on chromosome 5 was commonly identified in the control and PEG treatment groups and is associated with germination rate. The LRR kinase proteins are members of a larger family and several LRR proteins in Arabidopsis and rice. This family of proteins have been reported to be involved in seed germination, ABA and ROS signaling, and drought stress^54,55^. However, most of the reported LRR kinase genes in barley have been identified in response to pathogens^56–59^. The precise role of the identified LLR kinase in germination and drought response needs further investigation.

Flavin-containing monooxygenase add oxygen to lipophilic compounds or generate ROS. A tomato FMO1 protein physically interacted with catalase to modulate ROS and in turn elicited a drought tolerance response^60^. The Arabidopsis YUCCA6 gene was shown to harbor a novel FAD and NADPH-dependent-thiol reductase activity that aided in ROS regulation and conferred drought tolerance^61^. Recently, a maize YUCCA6 homolog and catalase were identified as hub genes in response to seedling drought stress^62^. Proteins containing tetratricopeptide repeat (TPRs) motifs are essential mediators of plant hormone signaling, such as signaling of gibberellic acid and ethylene, which act to form multiprotein complexes^63,64^. Polypeptides with TPRs may help mediate the plants stress response to drought. Based on these studies, the detailed functional analysis of the FMO and the TPR motif containing genes identified in response to PEG treatment is warranted.

Potassium which makes up of 2–10% of plant dry weight is essential for maintaining normal physiological and biochemical processes such as stomatal movement, photosynthesis, osmoregulation, protein synthesis, enzyme activation, and involved in the responses to biotic and abiotic stresses^65^. An adequate supply of K^+^ can increase the production of osmolytes such as proline^66^, reduce the production of toxic ROS by reducing the NADPH oxidase activity^67^, and increase water uptake under drought conditions. Osmo-priming effects of various potassium salts in improving seed germination pace and rate has been well known^68^. The interaction of protein kinase and calcium sensor proteins such as calcineurin-B-like protein leading to activation of potassium transporter for regulating K^+^ homeostasis has been reported in Arabidopsis^69^. The identification of exonic SNPs in a protein kinase, CBL protein and the K transporter in response to PEG treatment leads to speculate the existence of such a module in barley.

Seed germination is a process that involves the transition from cellular metabolic quiescence to an active metabolic state. DNA replication has been reported to start several hours after seed imbibition^70^. DNA polymerases are key enzymes for replication process, a fundamental event that precedes cell division and seedling establishment. DNA helicases are key enzymes for unwinding the DNA to enable the continuous replication of the leading and lagging DNA strands. Identification of an SNP in the PolII large subunit in this study in response to PEG treatment and in the greenhouse drought experiment suggests an important role for this gene during abiotic stresses.

DNA replication requires nucleotide pools to ensure active DNA synthesis. Inosine-5′-monophosphate dehydrogenase (IMPDH) catalyzes the NAD^+^-dependent oxidation of inosine monophosphate to xanthosine monophosphate, the rate-limiting step in the biosynthesis of guanine nucleotides. IMPDH is a regulator of the intracellular guanine nucleotide pool, and is therefore important for DNA and RNA synthesis, signal transduction, as well as other processes that are involved in cellular proliferation. Interestingly, not much is reported in the plant literature about IMPDH, except for an old report on this enzyme in pea seeds^71^ and warrants further investigation given their important role during cell proliferation.

ATP-dependent helicase with a bromo domain and plant homeodomain (PHD) finger containing protein are important readers of histone post-translational modifications. R3H domain containing protein have been reported to bind single stranded RNA or DNA^72^. A maize R3H domain containing protein (DIP1) has been shown to interact with drought responsive element (DRE) binding factor (DBF1)^73^. A rice R3H domain containing protein called OsMADS45-IP was shown to be induced in seeds and in vegetative tissues in response to cold, ABA, and osmotic treatment^74^. The role of these barley “post-translational readers” during germination and abiotic stress is certainly worth exploring.

Very long-chain fatty acids (VLCFAs) are essential precursors of membrane lipids, such as phospholipids and sphingolipids, cuticular waxes, suberins, and seed oils. The first step of VLCFA synthesis is mediated by 3-ketoacyl-CoA synthase (KCS), which catalyzes the condensation of a C2 unit from malonyl-CoA to acyl-CoA. Disruption of a KCS gene has been shown to impair root elongation in *Arabidopsis*^75^. In *Medicago truncatula*, a seed coat specific KCS was shown to be important for preserving physical seed dormancy^76^. Long-chain Acyl-CoA synthases (LACSs) are a subgroup of the ACS family that preferentially activate long-chain (C16-C18) or VLCFAs (< C20)^77^. In *Arabidopsis*, single and double mutants of the LACS were reported to show increased water loss and hypersensitivity to drought. Overexpressing the Apple LACS improved tolerance to PEG, salt, and ABA^78^. The KCS and LACS identified in this study could play a similar role in response to PEG-induced drought stress.

Group I, inositol polyphosphate phosphatases (5PTases) hydrolyze the water-soluble inositol polyphosphates, Ins(1,4,5)P3 and Ins(1,3,4,5)P4. The substrates for many *Arabidopsis* 5PTases have been identified^79^. Since several 5PTases could hydrolyze Ins(1,4,5)P3 to prevent its accumulation, it is hypothesized to terminate the corresponding Ins(1,4,5)P3 pathway and consequently modify abscisic acid (ABA) signaling, Ca^2+^ release, and ROS production^80–82^. In fact, two *Arabidopsis* 5PTases have been shown to be important for hydrolyzing inositol second-messenger substrates that impacts seed germination and early seedling development^83^. The induced expression of 5PTase only in the drought tolerant barely variety but not in the sensitive variety, and the nearly 5-fold repression in response to osmotic stress in Scarlett (drought-sensitive) cultivar (Supplementary Table 5) suggests an important role for this enzyme in mediating drought tolerance.

Succinate dehydrogenase assembly factors (SDHAF1) are involved in the insertion of Fe-S or FAD into the SDH proteins^84^. In *Arabidopsis*, SDHAF2 protein is required for assembly and activity of mitochondrial complex II and for normal root development^85^. Higher expression of this gene was recorded during early stages of seed germination, especially in the radicle (Supplementary Table 5). Identification of the SNP in the SDHAF1 associated with the dry root weight phenotype suggests that this gene could be playing a similar role in barley.

Copper-ion binding mitochondrial protein also annotated as At2g27730-like protein is a mitochondrial ATPase inhibitor, IATP. ATP synthase inhibitor prevents the enzyme from switching to ATP hydrolysis during collapse of the electrochemical gradient caused by stressful conditions such as the osmotic stress imposed by PEG. It supposedly inhibits ATP synthesis by preventing the release of ATP. One of the early cellular changes occurring during the early stages of germination is higher respiratory activity owing to the demands of ATP required for growth. One of the upshots of this increased respiration is the generation of excess ROS that can be detrimental to the germination process if it is not regulated. Mn/Fe SOD localized in the mitochondria has been shown to have very high expression during increased respiration^86^ and time-course expression data supports the higher transcript levels during very early stages of germination (Supplementary Table 5). Intrinsically higher levels of transcripts coding for antioxidant enzymes are key factors for imparting tolerance to abiotic stresses in barley^87,88^.

## Conclusions

The current study is the first report investigating the natural variation of barley genotypes to PEG-induced drought stress in seedling stage alongside short-term drought stress during heading in greenhouse conditions. Nine of the lines in the mini-core collection were unaffected under PEG-induced osmotic stress (100% germination). Two barley lines among this unaffected group of nine also exhibited tolerance to drought stress during heading stages. These lines could be useful resources for incorporation into drought tolerance breeding programs. The observed significant positive correlations and common GWAS loci across treatments for root traits indicate that selection for higher phenotypic values from the control conditions could serve as proxy for selecting genotypes with lower yield losses under drought stress. Co-identification of genic regions found by GWAS with representative growth stage dependent gene expression data from public repositories allowed for further filtering of potential candidate genes controlling germination, yield, and related traits across the control and drought stress groups. Based on the enrichment of the gene ontology for redox homeostasis, exploring the genetic regulation of oxidative signaling during germination under drought is an exciting area for further research. The identified favorable loci for drought tolerance could be exploited for marker assisted selection to support barley abiotic stress improvement breeding programs.

## Materials and methods

### Barely minicore panel

A diversity panel of 164 spring barley accessions were used in this study. This collection is a sub-sample of the larger USDA barley iCore panel^34^ selected with the goal of capturing maximum variability in the genome^15^. Our population is a subset of the USDA informative Core (iCore) panel which contains 1860 genotypes. Since our population is a miniature population representative of the iCore, we named our population the miniature iCore (mini-core). The mini-core population captures most of the allelic diversity present in its parent population, previously published^28^. Seeds for the current study were collected from the 2019 and 2020 field grow outs of the mini-core population in the West Madison Field Station, Madison, WI. The experiment complied with relevant institutional, national, and international guidelines and legislation.

### Germination traits

To evaluate genotypes’ germination performance, 10 seeds were selected for each of three technical replications for two biological replications. Seeds were then placed on Whatman grade 40 filter paper in 9-cm diameter glass Petri plates, followed by an additional top layer of filter paper. To start germination, filter paper was wetted with either 4mL of millipore water (control group) or a solution of PEG-6000 in a concentration of 20% (w/v) (PEG-treatment group) with osmotic potential at -1.09Mpa (VAPRO Vapor Pressure Osmometer). Petri plates with germinated seeds were stored in a cool and dry environment at room temperature (25ºC). Germination was defined when the leading radicle reached 2 mm in length, measured using a digital caliper (Model: Absolute AOS Digimatic, Mitutoyo) (Supplementary Fig. 1). Seeds were evaluated for germination at 24-hour intervals for five consecutive days. For seed germination, two phenotypes were recorded - germination percentage and germination rate (Table 1). These observations then used to calculate four derived traits - drought tolerance index of germination rate, drought tolerance index of germination percentage, reduction in germination percentage, and reduction in germination rate (Table 1).

### Seedling traits

To assess seedling development, five seeds were selected for each of three technical replications for two biological replications per genotype. Seeds were then placed on 38# regular weight 10” x 15” germination paper and rolled into tubes. Tubes were placed in 1 L beakers half filled with either millipore water or 20% (w/v) PEG solution, based on treatment group. Beakers with developing seedlings were stored in an incubation chamber on a 12-hour day/12-hour night cycle at 25 ºC. After seven days, root and shoot lengths were measured using a scaled ruler. The most representative seedling per treatment group was selected for image analysis. Representative seedlings were imaged, scanned, (Model: EPSON perfection STD4800 scanner, Regent Instruments) and analyzed using WinRHIZO software (Windows Version 7.6.8, Regent Instruments) for length, area, surface area, average diameter, and volume. Then, fresh weights were obtained using a sensitive balance (Mittler Toledo). Root and shoot tissues were dried for three days at 70ºC for dry weight measurements. In total, eight seedling-related traits were scored for shoots and roots separately - fresh weight, dry weight, total length, length, area, surface area, volume, and average diameter (Table 1).

### Drought stress screening in greenhouse

Four pots per accession were grown in the University of Wisconsin greenhouse facility. The soil mix, growing conditions such as light, temperature, and humidity in the greenhouse were as described earlier^46^. When the head from the main tiller emerged (~ 50% out of the boot), irrigation was withheld for five days. Soil moisture levels were recorded every day during these 5-days using a soil moisture probe (Moisture Meter HH2, Delta-T Devices, Cambridge, England). In the droughted plants the moisture levels were approximately 12–15% on days 4 and 5, when compared to the moisture levels in the well-watered controls. On day 6, plants were set for auto-irrigation until physiological maturity stage. Grain heads were harvested, threshed, and then weighed for yield. Along with seed yield, the dry shoot weight, and root weights for each plant was recorded (Table 1). The mini-core collection was subjected to drought screening in the years 2019 and 2020.

### Genotyping

Diversity panel was genotyped using the 50k Illumina™ Infinium iSelect SNP array^89^. This chip seq assayed a total of 44,040 SNPs and were aligned to *H. vulgare* Morex v3 reference genome^90^ for association analysis. The genotype data for the mini-core is deposited in the T3 database (https://triticeaetoolbox.org/barley/).

### Phenotypic trait analysis

Analysis of Variance (ANOVA) was conducted for seed and seedling traits to compare performance under control and PEG-induced drought conditions using RStudio.

Broad sense heritability was calculated in RStudio as H^2^ = $\:{{\sigma\:}_{g}^{2}/(\sigma\:}_{g}^{2}+\frac{{\sigma\:}_{g}^{2}}{Env.})$.

$\:{\sigma\:}_{g}^{2}$ is genotype variance and Env is the environment^91^.

### Correlation analysis

For the 155 genotypes, a Pearson’s phenotypic correlation analysis was performed under each of the experimental treatments. The correlation plots were constructed using the RStudio package “corrplot”^92^.

### Transformation of data

Residual Maximum Likelihood (REML) was used to analyze the phenotypic data of the accessions, while the Best Linear Unbiased Estimates (BLUEs) were calculated to estimate the mean of each trait for each accession and treatment using RStudio.

### Marker-trait association analysis (GWAS)

SNP filtering was performed on TASSEL5 for a minor allele frequency (MAF) ≥ 0.05. Using the estimated phenotypic traits (BLUEs) and SNP genotyping data, Fixed and random model Circulating Probability Unification Model (FarmCPU) was performed to determine marker-trait associations in RStudio in the package GAPIT. The FarmCPU model with four principal components generated the required kinship matrix to minimize the likelihood of false positive associations. Markers with a threshold P-value of 2 × 10^− 5^ (-log_10_*P* ≥ 4.65) which was set by association false discovery rate (FDR) of 0.01 were considered as associated makers^93^. Linkage Disequilibrium (LD) decay patterns level ≥ 0.1 identified a 1 Mb window surrounding significant markers for gene retrieval. Genes within this region (0.5Mbp flanking the significant SNP) were considered as putative candidates. SNPs located in genic regions and within a few kilobases to the predicted gene co-ordinates were considered as candidate genes. Functional annotations for the candidate genes were retrieved using the BARLEX database (http://apex.ipk-gatersleben.de) and the updated version 3 of the Morex genome in the Ensembl database (http://plants.ensembl.org/Hordeum_vulgare/Info/Index).

### Gene expression analysis

Gene expression data for the potential candidate genes were retrieved from the BarleyExpDB (http://barleyexp.com/index.html). Datasets (from Morex 3 set) associated with various developmental stages, seed germination, drought, and osmotic stress were selected. The accession numbers associated with these datasets are PRJEB1439, PRJEB40905, PRJNA261456, PRJNA27510, PRJNA439267, PRJNA496380, PRJNA679445 and PRJEB12540. Differential expression was determined based on comparison of different developmental stages or based on differences in the reported FPKM values between the treatment and control samples.

## Electronic supplementary material

Below is the link to the electronic supplementary material.

Supplementary Material 1

Supplementary Material 2

Supplementary Material 3

Supplementary Material 4

Supplementary Material 5

Supplementary Material 6

Supplementary Material 7

Supplementary Material 8

## Data Availability

The seeds of the mini-core collection are available from the USDA National Small Grains Collection (NSGC). Genotyping data for the mini-core population is deposited in the T3 barley database (https://triticeaetoolbox.org/barley/). All the data collected for this study is available as supplementary files. Mention of trade names or commercial products in this publication is solely for the purpose of providing specific information and does not imply recommendation or endorsement by the U.S. Department of Agriculture. USDA is an equal opportunity provider and employer.
