# Supplementary material for: Association mapping unravels the genetic basis for drought related traits in different developmental stages of barley
Source: Sci Rep. 2024 Oct 24;14:25121. doi: 10.1038/s41598-024-73618-y (PMC11502909; doi:10.1038/s41598-024-73618-y)
Supplement: Supplementary file 1 — Supplementary Material 1 [file 41598_2024_73618_MOESM1_ESM.pdf]

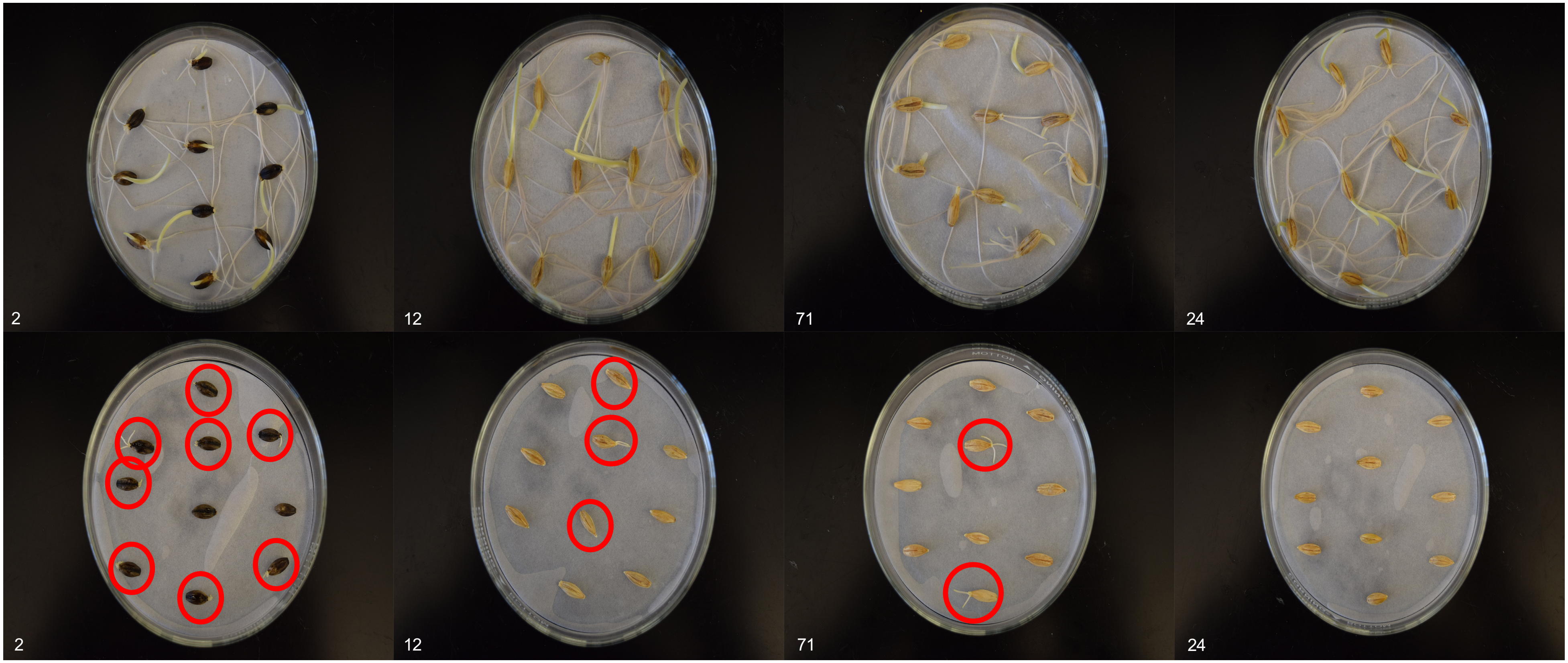

Supplementary figure 1: PEG-treatment phenotypes: Control: top panel, PEG treatment: bottom panel. Accession identification number in bottom left of image. Red circles denote germinated seeds in PEG treatment.
