# Supplementary material for: Association mapping unravels the genetic basis for drought related traits in different developmental stages of barley
Source: Sci Rep. 2024 Oct 24;14:25121. doi: 10.1038/s41598-024-73618-y (PMC11502909; doi:10.1038/s41598-024-73618-y)
Supplement: Supplementary file 2 — Supplementary Material 2 [file 41598_2024_73618_MOESM2_ESM.pdf]

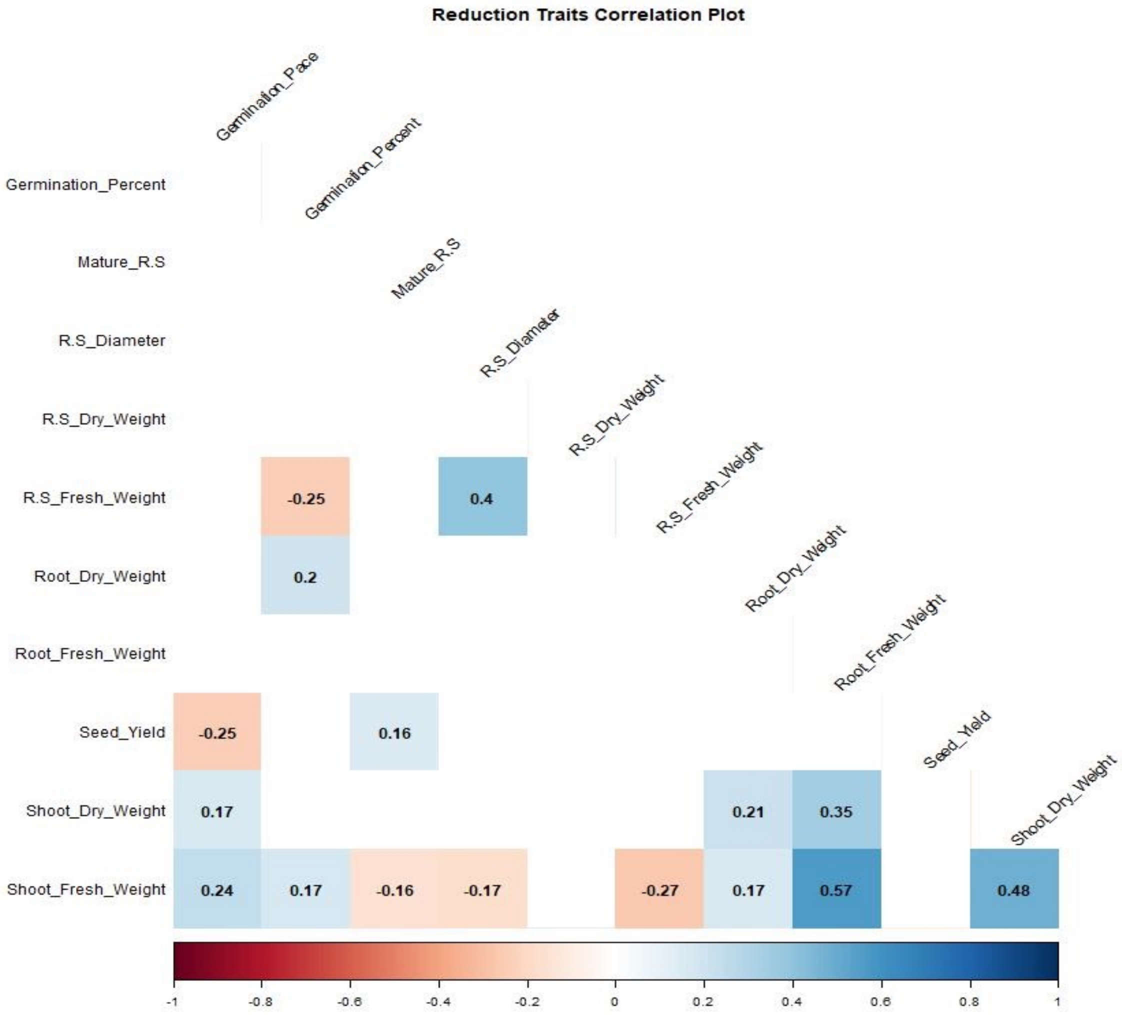

Supplementary Figure 2. Correlation analysis of the reduction traits associated with seed germination, seedlings, and adult plants in response to drought stress
