# Supplementary figures and images for: Association mapping unravels the genetic basis for drought related traits in different developmental stages of barley
Source: Sci Rep. 2024 Oct 24;14:25121. doi: 10.1038/s41598-024-73618-y (PMC11502909; doi:10.1038/s41598-024-73618-y)

# Illumina50k Minicore Marker Distribution

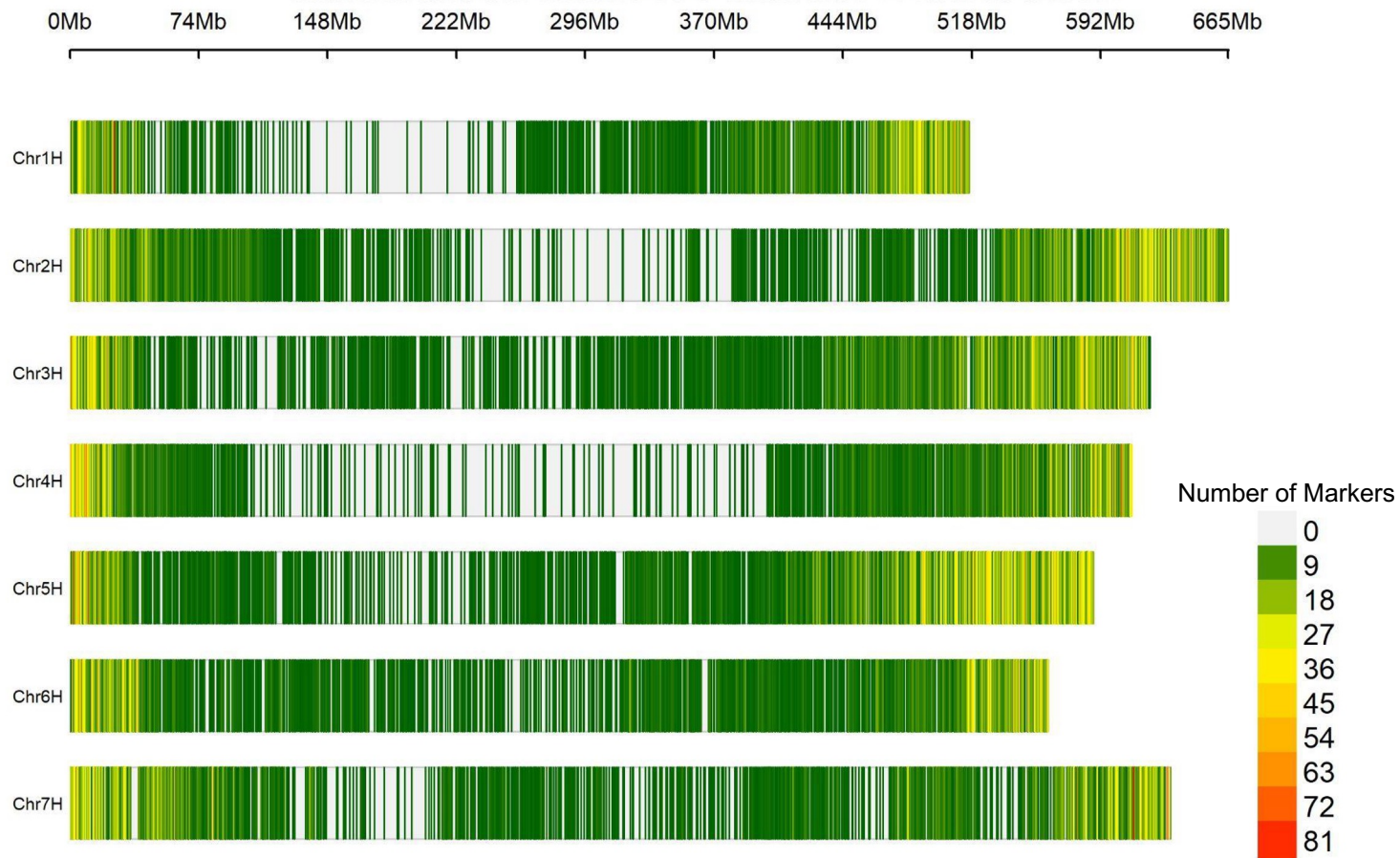

Supplement: Supplementary file 3 — Supplementary Material 3 [file 41598_2024_73618_MOESM3_ESM.pdf]
